# The Italian Network of Laboratories for Veterinary Oncology (NILOV) 2.0: Improving Knowledge on Canine Tumours

**DOI:** 10.3390/vetsci9080394

**Published:** 2022-07-30

**Authors:** Maria Ines Crescio, Giuseppe Ru, Luca Aresu, Elena Bozzetta, Maria Giovanna Cancedda, Katia Capello, Massimo Castagnaro, Azzurra Carnio, Cristiano Cocumelli, Barbara Degli Uberti, Claudia Eleni, Greta Foiani, Niccolò Fonti, Lucia Rita Gibelli, Lorella Maniscalco, Elisabetta Manuali, Valentina Moccia, Orlando Paciello, Antonio Petrella, Antonio Petrini, Alessandro Poli, Roberto Puleio, Elisabetta Razzuoli, Paola Scaramozzino, Katia Varello, Marta Vascellari, Valentina Zappulli, Angelo Ferrari, on behalf of NILOV

**Affiliations:** 1 Istituto Zooprofilattico Sperimentale del Piemonte, Liguria e Valle d’Aosta, Via Bologna, 148, 10154 Torino, Italy; giuseppe.ru@izsto.it (G.R.); elena.bozzetta@izsto.it (E.B.); elisabetta.razzuoli@izsto.it (E.R.); katia.varello@izsto.it (K.V.); angelo.ferrari@izsto.it (A.F.); 2 Dipartimento di Scienze Veterinarie, Università degli Studi di Torino, Largo Braccini, 2, 10095 Grugliasco, Italy; luca.aresu@unito.it (L.A.); lorella.maniscalco@unito.it (L.M.); 3 Istituto Zooprofilattico Sperimentale della Sardegna, Via Duca degli Abruzzi, 8, 07100 Sassari, Italy; mariagiovanna.cancedda@izs-sardegna.it; 4 Istituto Zooprofilattico Sperimentale delle Venezie, Viale dell’Università, 10, 35020 Legnaro, Italy; kcapello@izsvenezie.it (K.C.); gfoiani@izsvenezie.it (G.F.); mvascellari@izsvenezie.it (M.V.); 5 Department of Comparative Biomedicine and Food Science, University of Padua, Viale dell‘Università, 16, 35020 Legnaro, Italy; massimo.castagnaro@unipd.it (M.C.); valentinamoccia1993@gmail.com (V.M.); valentina.zappulli@unipd.it (V.Z.); 6 Istituto Zooprofilattico Sperimentale delle Regioni Lazio e Toscana, Via Appia Nuova, 1411, 00178 Roma, Italy; azzurra.carnio-esterno@izslt.it (A.C.); cristiano.cocumelli@izslt.it (C.C.); claudia.eleni@izslt.it (C.E.); paola.scaramozzino@izslt.it (P.S.); 7 Istituto Zooprofilattico Sperimentale del Mezzogiorno, Via Salute, 2, 80055 Portici, Italy; barbara.degliuberti@izsmportici.it; 8 Dipartimento di Scienze Veterinarie, Università degli Studi di Pisa, Viale delle Piagge, 2, 56124 Pisa, Italy; niccolo.fonti@phd.unipi.it (N.F.); alessandro.poli@unipi.it (A.P.); 9 Istituto Zooprofilattico Sperimentale della Lombardia ed Emilia, Via Giovanni Celoria, 12, 20133 Milano, Italy; luciarita.gibelli@izsler.it; 10 Istituto Zooprofilattico dell’ Umbria e delle Marche, Via Gaetano Salvemini, 1, 06126 Perugia, Italy; e.manuali@izsum.it; 11 Department of Veterinary Medicine and Animal Production, Unit of Pathology, University of Naples Federico II, Via Federico Delpino, 1, 80137 Napoli, Italy; paciello@unina.it; 12 Istituto Zooprofilattico di Puglia e Basilicata, Via Manfredonia, 20, 71121 Foggia, Italy; antonio.petrella@izspb.it; 13 Istituto Zooprofilattico di Abruzzo e Molise, Via Campo Boario, 64100 Teramo, Italy; a.petrini@izs.it; 14 Istituto Zooprofilattico Sperimentale della Sicilia, Via Gino Marinuzzi, 3, 90129 Palermo, Italy; roberto.puleio@izssicilia.it

**Keywords:** tumours, cancer registry, case series, canine tumours, animal cancer registry

## Abstract

**Simple Summary:**

Advances in cancer research are crucial, and pet oncology can improve the knowledge in several ways. Dogs are not only models of specific naturally occurring tumours but can also be sentinels of environmental exposures to carcinogenic substances, as they share the same environment with their owners. The purpose of this work was to describe the data collected by The Italian Network of Laboratories for Veterinary Oncology in the first 9 years of activity (2013–2021) and to evaluate their potential epidemiological significance. Frequencies of tumour sites in dogs were described, analysed and compared, considering several risk factors (breed, sex, period and region of residence). These observations allowed us to highlight differences not only in the site of occurrence of some tumours but also to formulate hypotheses on the potential role of some risk factors, e.g., neutering/spaying or geographical location. In our opinion, the results of this study confirm the importance of initiating and consolidating animal cancer registration initiatives that would facilitate the possibility of conducting multicentric collaborative studies to deepen the knowledge of the epidemiology of tumours in dogs and, from a comparative perspective, in humans.

**Abstract:**

Advances in tumour research are crucial, and comparative oncology can improve the knowledge in several ways. Dogs are not only models of specific naturally occurring tumours but can also be sentinels of environmental exposures to carcinogens, as they share the same environment with their owners. The purpose of this work was to describe the data collected by The Italian Network of Laboratories for Veterinary Oncology in the first 9 years of activity (2013–2021) and to evaluate their potential epidemiological significance. Frequencies of tumour topographies and main morphologies in dogs were described, analysed and compared, calculating age-adjusted proportional morbidity ratios and considering several risk factors (breed, sex, period and region of residence). These observations allowed us to highlight differences not only in morphology and topography of some tumours but also to formulate hypotheses on the potential role of some risk factors, e.g., neutering/spaying or geographical location. In our opinion, the results of this case series confirm the importance of initiating and consolidating animal cancer registration initiatives that would facilitate the possibility of conducting multicentric collaborative studies to deepen the knowledge of the epidemiology of tumours in dogs from a comparative perspective.

## 1. Introduction

The Global Burden of Disease Study reported that between 2007 and 2017, incident tumour cases in people showed a significant increase, and, from 1990 to 2017, tumour raised from sixth to second place in the top causes of disability-adjusted life years (DALYs) loss [1]. Advances in tumour research are crucial and comparative oncology can improve the knowledge in multiple ways. Dogs represent valid animal models for several naturally occurring neoplasia including, but not limited to, urinary bladder tumours [2], osteosarcomas [3], and lymphomas [4,5], as they bear notable similarities to their human counterparts in terms of biological behavior, morphology, molecular features [6] and genetics [7,8]. Furthermore, as they share the same environment with their owners, dogs can be sentinels of environmental exposures to carcinogens, as reported in mesothelioma due to the exposure to asbestos [9] and testicular and bladder tumours due to exposure to herbicides [10,11] and insecticides [12].

Tumours are included by WHO in the group of non-communicable diseases, and epidemiological studies in humans and animals rely on tumour registration, with severe limitations due to the fragmentary communication of the data, particularly for animal cancer. The first canine tumour registry was established in the 1960s in the USA [13,14], and since then, several efforts to gather data on canine neoplastic processes have been made at different geographical levels, using tissue banks, animal insurance databases, hospital-based registries and population-based registries [15].

In Italy, the first population-based canine tumour registry was established in Genoa in 1985 [16], followed by several other registries [17,18,19]. However, the extension to the whole Italian territory is far from complete. The Italian Network of Laboratories for Veterinary Oncology (NILOV) was created in 2013 to collect into a single database the diagnoses of tumours in pets from multiple sources and also to facilitate collaboration. Initially, case-series data were collected only from the Veterinary Public Laboratories (Istituti Zooprofilattici Sperimentali, IIZZSS), then the network was gradually extended to involve four schools of veterinary medicine and several canine cancer registries from different Italian geographical areas (i.e., Verona and Vicenza, Lazio, Umbria and Marche, Campania, Abruzzo). In order to improve its completeness, further efforts are ongoing to widen NILOV’s coverage and to include pathologists from other Universities and private laboratories. Differently from population-based tumours registries, the Nilov data set is based on patient attendance at hospitals and clinics and is mainly constructed from laboratory-based surveillance, whereas the collection of data on the underlying populations is not in its remit.

The purpose of this work was to describe the data collected on canine tumours over a spanning period of 9 years (2013–2021) and to evaluate their potential epidemiological significance. To achieve this goal, frequencies of tumour topographies and main morphological diagnosis were described, analysed and compared, considering age, breed, sex, period and region of residence.

## 2. Materials and Methods

### 2.1. Data Collection and Handling

The NILOV database collects tumours data directly from the partner institutions. Data are uploaded via the web according to a shared record layout. All diagnoses are classified according to the WHO International Histological Classification of Tumours of Domestic Species [20,21,22,23,24,25,26,27,28,29,30,31,32,33] and coded based on morphology and topography, using appropriately adapted ICD-O and ICD-X classification systems [34] agreed upon among pathologists. Individual information on dogs’ breed, sex, neutered/spayed status, date of birth, date of diagnosis, national territorial unit code of the town of owner’s residence and an alphanumeric string uniquely identifying the owner’s surname are also collected. Submitting owners and vets signed in each Institution an informed consent for privacy and to allow the use of anonymised protected data regarding samples in research studies. Institutional ethical approval was not required.

For the current study, age at diagnosis was obtained by subtracting the date of birth from the date of diagnosis and categorised into seven age classes (0–3 years, 4–5 years, 6–7 years, 8–9 years, 10–11 years, 12–13 years, and 14 years and more). Breeds were furtherly classified into two groups (purebred, not purebred).

Based on the Nomenclature of Territorial Units for Statistics, Italian regions are grouped according to the NUTS1 level into 5 macro-regions (North-East, North-West, Center, South and Islands). For the purposes of this work and on the basis of geographical, historical, cultural and economic similarities, data from the North-East were combined with that from the North-West and those from the South with those from the Islands. Based on the date of the first diagnosis, data were arbitrarily divided into two time periods: from January 2013 to December 2017 and from January 2018 to December 2021.

Diagnoses were grouped according to Gruntzig et al. [35] to investigate malignancy; each tumour group was divided into benign (behaviour code 0–2) and malignant (behaviour code 3–6) according to the ICD-O classification.

The tumour location was obtained by grouping the topographical codes into 15 groups according to Gruntzig et al. [35], except for tumours of peripheral nerves and autonomic nervous tissues that were grouped together with the soft tissue tumours (see Appendix B, Table A1). Based on their location, tumours were further classified as either external (mammary gland; skin; male sexual organs) or visceral (bones, joints, cartilage; eye, brain, meninges; endocrine glands; gastrointestinal tract; other female sex organs; respiratory system, intrathoracic organs; retroperitoneum, peritoneum; soft tissues; urinary organs).

In the case of repeated records for the same tumour in the same location in the same animal, only one tumour event was considered. If the same animal was diagnosed with more than one tumour type or location, these were recorded as separate events. Metastatic tumours were excluded from the analysis.

### 2.2. Data Analysis

A descriptive analysis of the data was carried out, taking into consideration age at first diagnosis, sex, neutered/spayed status, region of residence and tumour location.

In the absence of suitable denominators, proportional morbidity (PM) and relative 95% confidence intervals (95% CI) were used as a measure of occurrence. PM is the proportion of cases for a specific category of the tumour over the total of the tumours (e.g., the number of malignant mammary tumours over the total number of malignant tumours, where the tumour location is intended as the category of interest); each specific category is expressed as a percentage of all tumours, and the sum of the categories must add to 100%. PM ratios (PMR) were used to make comparisons between groups (e.g., spayed vs. not spayed females), origin, or time periods and it was interpreted as a proxy of relative risks. In our study, PMR was calculated as the ratio between the PM of two different populations with different exposure (e.g., PM of malignant mammary tumours in spayed females/PM of malignant mammary tumours in not spayed females, where the category is the tumour location, and the two compared populations are spayed/not spayed females) [36,37,38].

PMR were obtained by means of Poisson regression models to assess the risk of tumour category by the available covariates (e.g., sex and neutered status, or being purebred or mixed, or time period or macro-region, etc.) and were adjusted by age-class. PMR for time period and for macro-region were also adjusted for the breed (purebred vs. mixed) and neutering status.

Interactions between space and time for visceral and external tumours were assessed graphically.

All statistical analyses were carried out using STATA 17.0 (StataCorp, College Station, TX, USA).

## 3. Results

As the main point of cancer registration was the study of malignancies, for their impact on health, we focused our work on malignant tumours, reporting data on benign tumours in the appendix (Appendix C).

### 3.1. Individual Data

**Figure 1:**
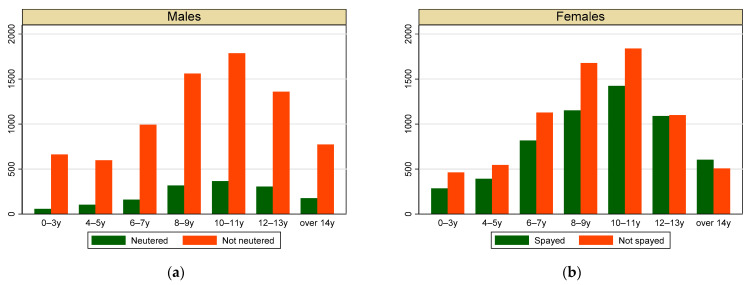
Distribution of dogs with a diagnosis of tumour by sex, neutered/spayed status and age class at first diagnosis. (**a**) Males; (**b**) females.

**Figure 2:**
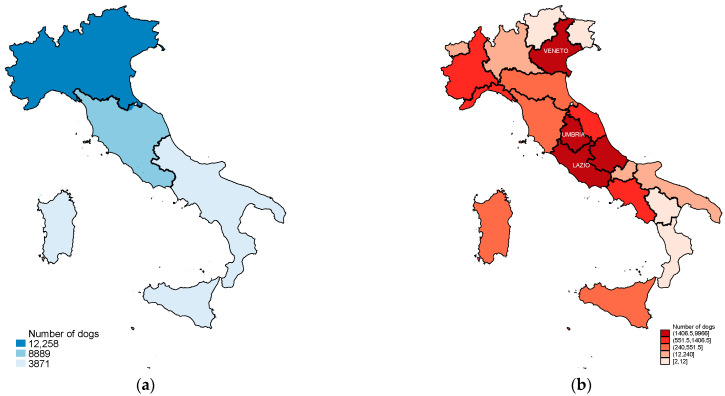
Choropleth maps showing the number of dogs included in the study. The gradient of colour is proportional to the frequency of observations: the darker the colour, the higher the frequency. (**a**) Absolute number of dogs with a diagnosis of tumour by macroregion of residency of the owner (North, Centre, South and Islands), (**b**) number of dogs with a diagnosis of tumour by Italian region, classes follow a quantile distribution, with regions grouped in 5 quintiles, with 4 Regions each. “()“ = excluded; “[]” = included.

During the first 9 years of activity, the NILOV database collected 28,727 diagnoses on 25,024 dogs. Most of the dogs were female (57%), and as shown in Figure 1, most of males (95%) and females (59%) were not neutered/spayed. The mean age at first diagnosis was similar in both sexes, with small differences in neutering status and cancer behavior: neutered/spayed dogs were slightly older at first diagnosis than entire ones, and malignant tumours were firstly diagnosed in slightly older dogs (Table 1). The most represented age classes were between 6 and 13 years, without differences between sexes (Figure 1). Over 41% of dogs were mixed breed; the most frequent breeds were German shepherd (4.73%), Labrador retriever (4.39%), Boxer (3.18%) and Pinscher (3.12%). As shown in Figure 2, most of the dogs came from the northern macro-region (Figure 2a); nevertheless, the regional distribution showed that we had cases covering the whole Italian territory and some regions, especially those that have had active registries for a long time (Veneto, Umbria, Lazio) were more represented than others (Figure 2b).

### 3.2. Malignant Tumours

**Figure 3:**
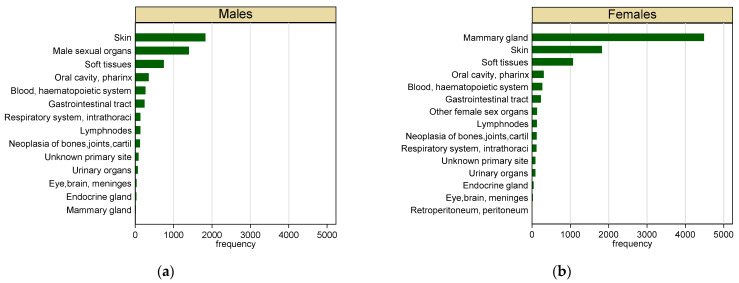
Frequency of malignant tumours in dogs by sex and topography; (**a**) males, (**b**) females.

**Figure 4:**
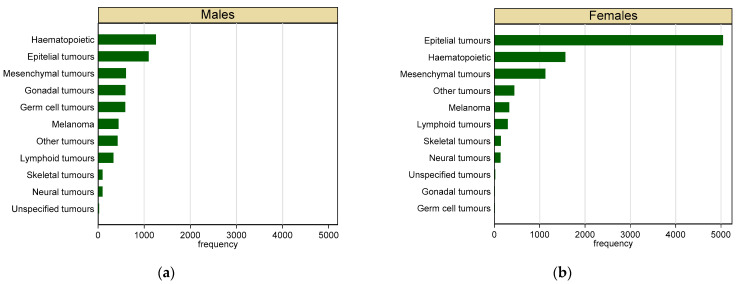
Frequency of malignant tumours in dogs by sex and tissue type. (**a**) Males; (**b**) females.

Malignant tumours represented 53% of the total cases collected (n = 15,083). Most of the malignant tumours (60%) occurred in females. As shown in Figure 3, skin (32%) and sexual organs (25%) were the most frequent location for malignant tumours in male dogs, whereas mammary gland (48%) and skin (19%) were the most frequent in females. Haematopoietic (22%) and epithelial (20%) were the most represented tumours in male dogs, whereas epithelial tumours (55%) and haematopoietic tumours (17%) were the most frequent in females (Figure 4).

**Figure 5:**
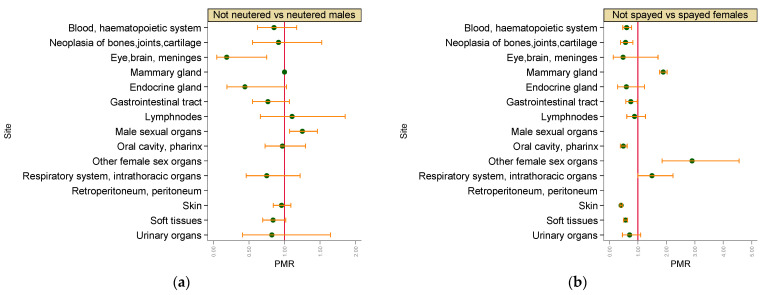
Proportional morbidity ratios of malignant tumours by topography: (**a**) neutered males vs. not neutered males, (**b**) spayed females vs. not spayed females. Dots: age-class-adjusted PMRs, bars: lower and upper bound of 95% confidence interval. A vertical reference line indicates PMR = 1. (**a**) Males; (**b**) females.

As shown in Figure 5, not neutered males, when compared with neutered ones, had a significantly increased age-adjusted risk of malignant tumours in male sexual organs (PMR = 1.25, 95% CI 1.07–1.46), whereas being not neutered seemed to decrease the risk of eye, brain and meninges malignant tumours (PMR = 0.19, 95% CI 0.05–0.75). Not spayed females, when compared with spayed ones, had significantly increased age-adjusted risk of malignant mammary gland (PMR = 1.89, 95% CI 1.77–2.03) and other females sexual organs (PMR = 2.90, 95% CI 1.85–4.56), whereas being not spayed seemed to have a protective effect for malignant tumours of blood, haematopoietic system (PMR = 0.61, 95% CI 0.47–0.78), bones, joints and cartilage (PMR = 0.57, 95% CI 0.39–0.83), gastrointestinal tract (PMR = 0.75, 95% CI 0.58–0.98), oral cavity and pharynx (PMR = 0.49,95% CI 0.38–0.62), skin (PMR = 0.41, 95% CI 0.37–0.45) and soft tissues (PMR = 0.57, 95% CI 0.50–0.64).

**Figure 6:**
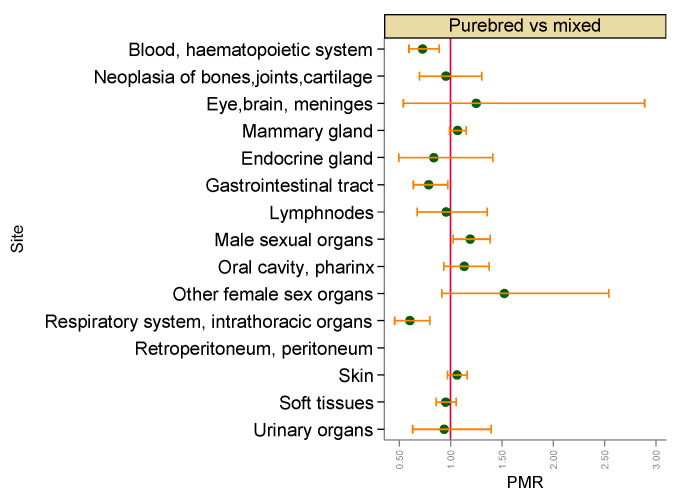
Proportional morbidity ratios of malignant tumours by topography comparing purebred dogs with mixed dogs. Dots: age-class-adjusted PMRs, bars: lower and upper bound of 95% confidence interval. A vertical reference line indicates PMR = 1.

The comparison between purebred and mixed dogs showed for purebred dogs a slight but statistically significant increased age-adjusted risk of malignant tumours in male sexual organs, whereas being purebred showed a protective effect on malignant tumours of blood and haematopoietic system (PMR = 0.73, 95% CI 0.60–0.89), gastrointestinal tract (PMR = 0.79, 95% CI 0.64–0.97) and respiratory system and intrathoracic organs (PMR = 0.60, 95% CI 0.46–0.80) (Figure 6).

**Figure 7:**
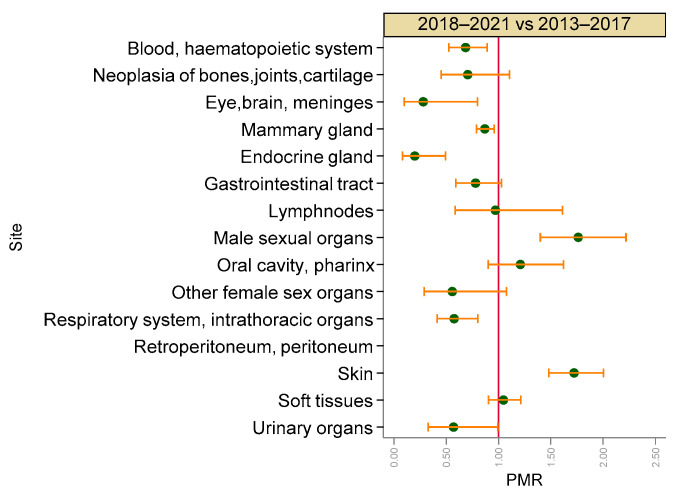
Proportional morbidity ratios (PMR) of malignant tumours by topography comparing the last years of activity (2018–2021) with the first period of activity (2013–2017). Dots: age-class-adjusted PMRs, bars: lower and upper bound of 95% confidence interval. A vertical reference line indicates PMR = 1.

Compared with previous years (2013–2017), the last years of activity (2018–2021) showed a significantly increased age-, breed- (purebred or mixed) and neutering-status-adjusted risk of malignant tumours of male sexual organs (PMR = 1.76, 95% CI 1.40–2.22) and skin (PMR = 1.72, 95% CI 1.48–2.00), whereas the risk of malignant tumours of blood and haematopoietic system (PMR = 0.68, 95% CI 0.53–0.89), eye, brain and meninges (PMR = 0.28, 95% CI 0.10–0.80), mammary gland (PMR = 0.87, 95% CI 0.79–0.96), endocrine glands (PMR = 0.20, 95% CI 0.08–0.49), respiratory system and intrathoracic organs (PMR = 0.58, 95% CI 0.41–0.80), and urinary organs (PMR = 0.57, 95% CI 0.33–0.99) was significantly reduced (Figure 7).

**Figure 8:**
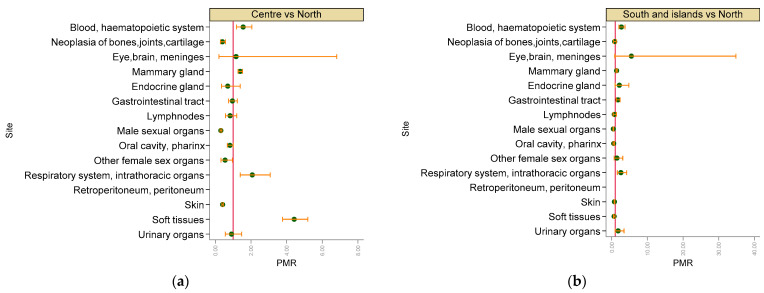
Proportional morbidity ratios (PMR) of malignant tumours by topography comparing the Italian macro regions ((**a**,**b**) North, Centre, South and islands) with the others. Dots: age-clas- adjusted PMRs, bars: lower and upper bound of 95% confidence interval. A vertical reference line indicates PMR = 1.

The risk for malignant tumours in a specific location showed striking differences between the three Italian macro-regions (Figure 8).

Compared to the Northern Italian regions, central Italian regions showed a significantly increased age-, breed- (purebred or mixed) and neutering-status-adjusted risk of malignant tumours of blood and haematopoietic system (PMR = 1.56, 95% CI 1.19–2.05), mammary gland (PMR = 1.40, 95% CI 1.28–1.53), respiratory system and intrathoracic organs (PMR = 2.07, 95% CI 1.40–3.08) and soft tissues (PMR = 4.42, 95% CI 3.77–5.19) compared to Northern Italy, whereas there was a reduced risk of malignant tumours of bones, joints and cartilage (PMR = 0.40, 95% CI 0.28–0.57 male sexual organs (PMR = 0.31, 95% CI 0.26–0.36), other female sexual organs (PMR = 0.55, 95% CI 0.326–0.95) and skin (PMR = 0.41, 95% CI 0.37–0.45).

Compared to the Northern Italian regions, Southern Italian regions and islands showed significantly increased age-, breed- (purebred or mixed) and neutering-status-adjusted risks of malignant tumours in blood and hematopoietic system (PMR = 2.73, 95% CI 2.004–3.73), mammary gland (PMR = 1.37, 95% CI 1.22–1.54), gastrointestinal tract (PMR = 1.76, 95% CI 1.31–2.38), respiratory system and intrathoracic organs (PMR = 2.57, 95% CI 1.57–4.21), whereas there was a significantly reduced risk of malignant tumours of male sexual organs (PMR = 0.46, 95% CI 0.37–0.57), oral cavity and pharynx (PMR = 0.57, 95% CI 0.40–0.80), skin (PMR = 0.77, 95% CI 0.68–0.87) and soft tissues (PMR = 0.68, 95% CI 0.50–0.93).

**Figure 9:**
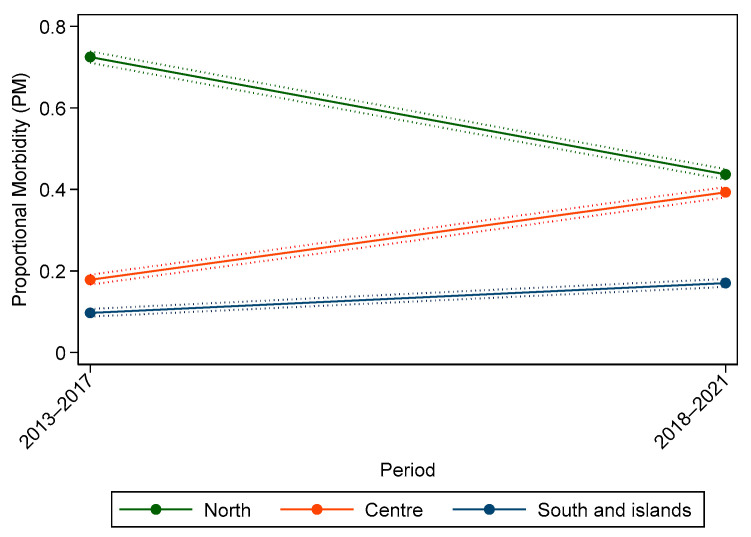
Proportional morbidity (PM) of external and visceral malignant tumours by macroregions (solid lines) and their 95% confidence interval (dotted lines).

Figure 9 reports proportional morbidity (PM) of visceral malignant tumours in different periods and macroregions, showing a decrease in PMs in Northern Italy, a clear increase in PMs in Centre Italy and a smaller increase in South and islands. As the trends were not parallel, the effect of time was modified by the macroregion of residence; therefore, there is suggestion of interaction between space and time.

## 4. Discussion

Our study allowed us to not only confirm differences in histotype and topography of some tumours by malignancy and sex but also identify some differences in reproductive status and geographical location.

The ICD-O codes used for the collection of data were not specific for veterinary entities because, at the time of the data collection, the Vet-ICD-O-Canine-1 [39] was not available; therefore, it was not applied. However, the adapted ICD-O and ICD-X classification systems that were applied by the pathologists of our network were preliminarily agreed upon and allowed the application of a harmonised system of classification and efficient transmission of unambiguous data. Moreover, in the frame of data analysis, we traced the morphological codes to the higher hierarchically groups to overcome potential differences in code assignment by the individual pathologists.

Potential misclassification of diagnosis may still be present: however, agreement studies are periodically (annually) carried out within the network showing that the agreement on diagnostic codes is satisfactory (data not published).

As no data on reference population (denominators) were available and the collection of tumours (numerators) was non-exhaustive, we were not able to calculate incidence data of cancer but only to deal with proportions. Therefore, to compare data and to calculate measures of association, we calculated proportional morbidity risks (PMR). PMR can be considered a valuable tool for exploratory analysis but should be interpreted cautiously, as their denominator is the total number of cases and not the population at risk; therefore, we can’t assure full external validity [36]. Furthermore, the PMRs allow us to directly compare our results with those reported in literature only when the latter are reported in the form of proportions. Age at first diagnosis was similar to the findings reported by Pinello et al. in Portugal [40] in both sexes and of Bronden et al. in Denmark [41] for females, whereas males were slightly older in our study. As the incidence of most canine tumours increases with age [16,17,18,35,41], all our estimates were adjusted by age class.

As reported by several studies [16,17,18,41], female dogs develop neoplasia more frequently than male dogs; however, this finding seems to be the consequence of the higher occurrence of sex-specific tumours and cannot be generalised to all tumours. The proportion of neutered/spayed dogs is clearly lower than that of entire ones; this is particularly evident in male dogs. The differences between Europe and USA in neutering/spaying habits have been claimed as the reason for differences between the female/male ratio in cancer development in dogs’ cancer registries located in different geographical areas [16]. Our results showed a higher risk for sex-specific tumours in not neutered or not spayed dogs, but a protective effect in benign and malignant tumours of the skin, soft tissue, oral cavity and pharynx. Further targeted studies are recommended to investigate the effect of neutering/spaying on specific tumours morphologies, such as hemangiosarcoma, osteosarcoma, lymphoma, transitional cell carcinoma and mast cell tumours [42,43].

The comparison of data on histotype is complex due to the different aggregation levels and indexes reported: the proportion of malignant tumours is slightly superior to that of benign tumours, consistently with data previously reported in Italy [16,17,18] and in Europe [35,39,41], whereas Dobson et al. [44] report a relevant higher proportion of benign tumours in the UK. In our study, small differences in the most common histotypes were seen between benign and malignant tumours and for malignant tumours also between sexes. To our knowledge, a similar distinction was not previously reported in the literature, where data on morphology indicates a higher general proportion of epithelial and mesenchymal tumours, with a higher proportion of mammary tumours [35,41].

Most of the published studies, with minimal differences, indicates mammary gland tumours in females and skin in males as the most frequent location for tumours [17,18], whereas when no sex distinction was made, skin, mammary gland and soft tissues were the most common location of tumours [35,41]. Mammary gland, genital and skin tumours are indeed easier to recognise by the owner and by physical examination by the practitioner than tumours of internal organs, which require specific and more expensive diagnostic imaging exams.

The comparison of periods, adjusted by age, breed and neutering status, showed significant increased risks of male sexual organs and skin tumours, but a decreased risk in several sites including mammary gland. Few studies in the literature report changes in the incidence of cancer during the years [45] but do not compare periods in terms of risk, so our data are not comparable.

Significant differences were also found between the geographical distribution of tumours, confirmed by the interaction of space and time, showing that the effect of time is modified by the macroregion of residence. Those results could be influenced by the different levels of case detection implemented during the time in the different Italian regions and could reflect more a change in the data collection due to several factors, e.g., greater perception of the importance of tumour registration by practitioners and owners, improvement in diagnostics, more than a real change in tumours’ risk factors.

These differences must be investigated through ad hoc inter or intra registry studies at more detailed geographical level and with a well-defined underlying population.

## 5. Conclusions

Our study allowed a collection of numerous data on canine tumours from several Institutions located throughout the whole Italian territory. Despite some limitations, this is very relevant to broaden the knowledge on cancer epidemiology and, therefore, possible risk factors also in a comparative perspective. With this study, we remark on the importance of initiating and consolidating cancer registration initiatives that would facilitate the conduct of multicentric collaborative studies and would allow a progressive decrease in biases in data collection.

## Figures and Tables

**Table 1:** Mean age at first diagnosis by sex, neutering status and tumour behavior.

Sex and Neutering Status	Mean Age at Diagnosis (Years) Benign	Mean Age at Diagnosis (Years) Malignant
Male, neutered	9.6	10.1
Male, not neutered	8.8	9.6
Female, spayed	9.2	9.9
Female, not spayed	8.6	9.4

**Figure A1:**
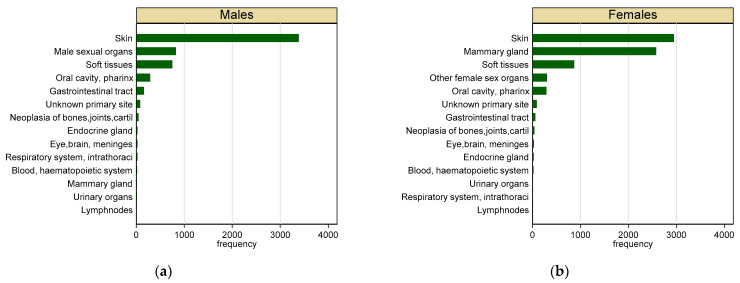
Frequency of benign tumours by sex and topography. (**a**) Males; (**b**) females.

**Figure A2:**
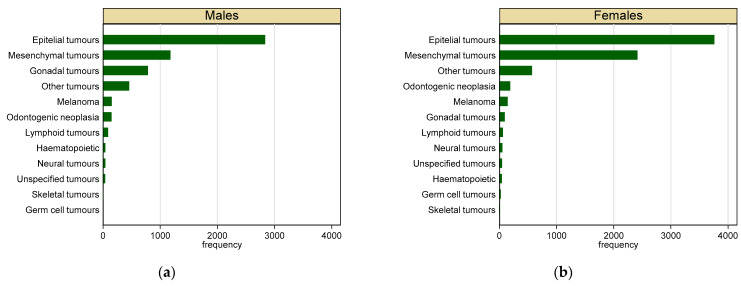
Frequency of benign tumours by sex and histotype. (**a**) Males; (**b**) females.

**Figure A3:**
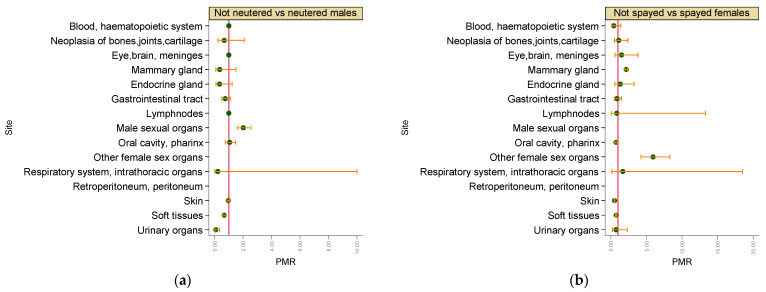
Proportional morbidity ratios (PMR) of benign tumour by topography: (**a**) not neutered males vs. neutered males, (**b**) not spayed females vs. spayed females. Dots: age-class-adjusted PMR; bars: lower and upper bound of 95% confidence interval. A vertical reference line indicates PMR = 1.

**Figure A4:**
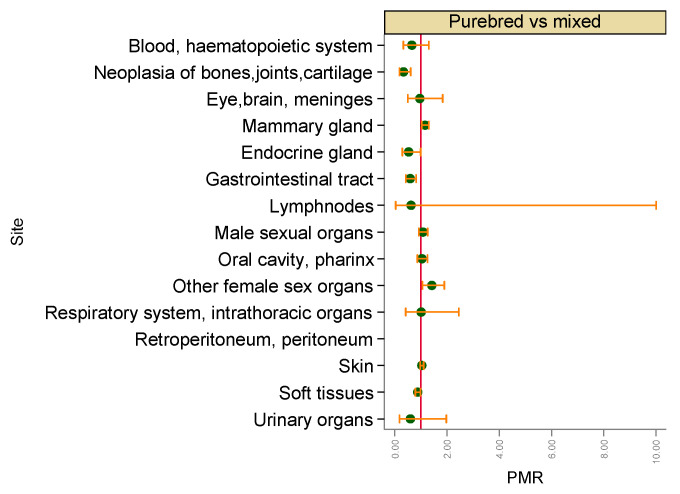
Proportional morbidity ratios (PMR) of benign tumours by topography comparing purebred and mixed dogs. Dots: age-class-adjusted PMR, bars: lower and upper bound of 95% confidence interval. A vertical reference line indicates PMR = 1.

**Figure A5:**
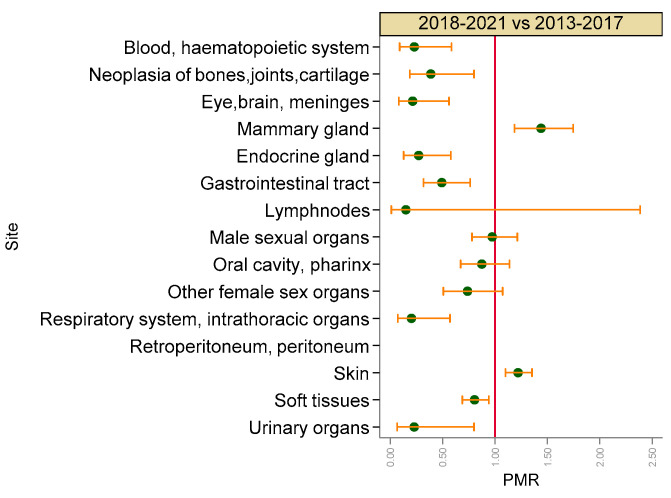
Proportional morbidity ratios (PMR) of benign tumours by topography comparing benign tumours diagnosed in the last years of activity (2018–2021) with the first period of activity (2013–2017). Dots: age-class-adjusted PMR, bars: lower and upper bound of 95% confidence interval. A vertical reference line indicates PMR = 1.

**Figure A6:**
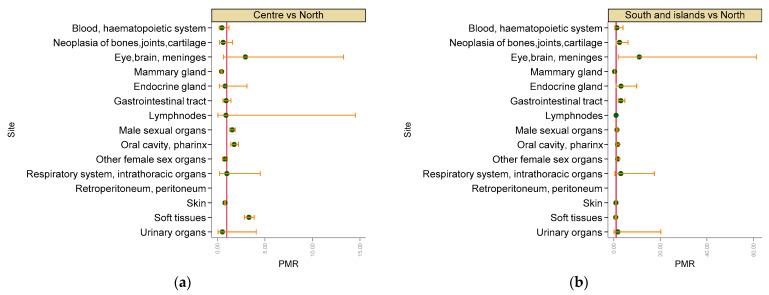
Proportional morbidity ratios of benign tumours by topography comparing benign tumours diagnosed in Centre (**a**) and South and Island (**b**) with Northern Italian regions. Dots: age-class-adjusted PMR, bars: lower and upper bound of 95% confidence interval. A vertical reference line indicates PMR = 1.

**Figure A7:**
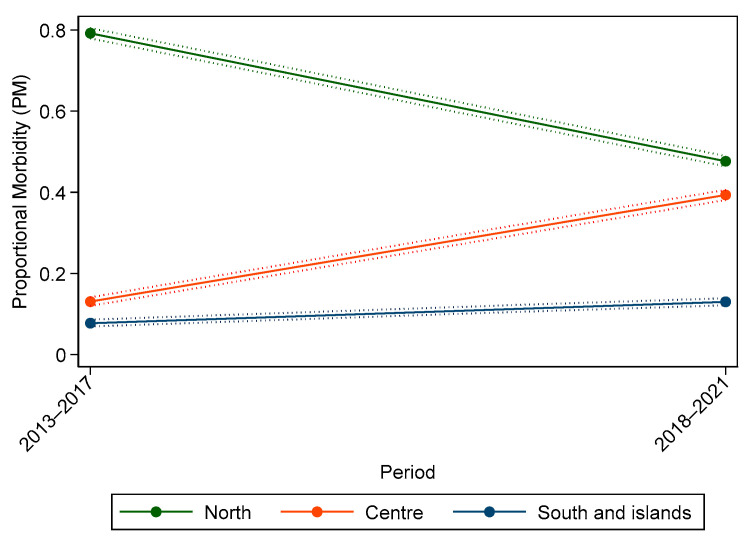
Proportional morbidity (PM) (solid lines) and their 95% confidence interval (dotted lines) of visceral benign tumours by period and macroregions.

## Data Availability

Data are available on request.

## Appendix A. NILOV Contributors

Paola Modesto, Marzia Pezzolato, Alessandra Ratto, Lisa Guardone (Istituto Zooprofilattico Sperimentale del Piemonte, Liguria e Valle d’Aosta).

Silvia Ferro, Laura Cavicchioli, Ranieri Verin (Department of Comparative Biomedicine and Food Science, University of Padua).

Claudia Zanardello, Antonio Carminato (Istituto Zooprofilattico Sperimentale delle Venezie).

Claudio Pigoli (Istituto Zooprofilattico Sperimentale della Lombardia e dell’Emilia romagna).

Sabrina Vanessa Patrizia Defourny, Giovanni Di Teodoro, Daniela Malatesta ((Istituto Zooprofilattico Sperimentale dell’Abruzzo e del Molise).

Silvia Pavone (Istituto Zooprofilattico Sperimentale dell’Umbria e delle Marche).

Serenella Papparella, Valeria Baldassarre (Department of Veterinary Medicine and Animal Production, Unit of Pathology. University of Naples Federico II).

## Appendix B

**Table A1:** Coding of tumour locations according to Grunzig et al. [35].

Location	ICD-9	ICD-10
Blood, haemopoietic system	T 169.0–169.9	C 42
Neoplasia of bones, joints, cartilage	T 170.0–170.9	C 40–41
Brain, meninges, other parts of CNS	T 190.0–192.9	C 70–72
Mammary gland	T 174.0–175.9	C 50
Endocrine gland	T 193.0–194.9	C 73–75
Gastrointestinal tract	T 150.0–159.9 (158 excluded)	C 16–26.8
Lymph nodes	T 196.0–196.9	C 77
Male sexual organs	T 185.0–186.9; T 187.1–187.9	C 60–63.2
Oral cavity, pharynx	T 140.0–149.9	C 2.9–11
Other female sex organs	T 179.0 -184.9	C 51–58
Respiratory system, intrathoracic organs	T 160.0–165.9	C 30–39
Retroperitoneum, peritoneum	T 158	C 48
Skin	T 173.0–173.9	C 44
Soft tissues	T 171.0–171.9	C 49; C47
Urinary organs	T 188.0–189.9	C 67–68

## Appendix C

### Results Regarding Benign Tumours

Benign tumours represented the 47% of the total collected cases (n = 13,553). Most of benign tumours occurred in females (55%). As shown in Figure A1, in male dogs, skin (59%) and male sexual organs (14%) were the most frequent locations, whereas in females, the most frequent locations were skin (39%) and mammary gland (35%). Epithelial (49%) and mesenchymal benign tumours (20%) were the most represented in male dogs, as well as in females, where epithelial tumours represented the 51% and mesenchymal benign tumours the 33% (Figure A2).

As shown in Figure A3, not neutered males were more at risk of carrying male sexual organs benign tumours (PMR = 2.02, 95% CI 1.59–2.56), whereas being not neutered seemed to have a protective effect on benign tumours of soft tissues (PMR = 0.68, 95% CI 0.56–0.81) and urinary organs (PMR = 0.09, 95% CI 0.02–0.34). Similarly, not spayed females had a significantly increased age-adjusted risk of benign tumours of the mammary gland (PMR = 2.16, 95% CI 1.97–2.36) and other female sexual organs (PMR = 5.93, 95% CI, 4.24–8.29), whereas not spaying seemed to have a protective effect on benign tumours of oral cavity and pharynx (PMR = 0.72, 95% CI 0.56–0.91), skin (PMR = 0.52, 95% CI 0.48–0.56) and soft tissues (PMR = 0.75, 95% CI 0.65–0.86).

Purebred dogs, compared with mixed ones, showed an increased age-adjusted risk of benign tumours of mammary gland (PMR = 1.16, 95% CI 1.04–1.31) and other female sexual organs (PMR = 1.42, 95% CI 1.06–1.90), whereas being purebred showed a protective effect on benign tumours of bones, joints and cartilage (PMR = 0.34, 95% CI 0.18–0.61) and gastrointestinal tract (PMR = 0.59, 95% CI 0.43–0.82) (Figure A4).

During the last years (2018–2021), there was an increased age-, breed- and neutering status-adjusted risk of diagnosing benign tumours of the mammary gland (PMR = 1.44, 95% CI 1.19–1.75) and skin (PMR = 1.22, 95% CI 1.10–1.35), whereas there was a significative reduced risk of diagnosing benign tumours of blood and haemopoietic system (PMR = 0.23, 95% CI 0.09–0.58), neoplasia of bones, joints and cartilage 8PMR = 0.39, 95% CI 0.19–0.80), eye brain and meninges (PMR = 0.21, 95% CI 0.08–0.56), endocrine glands (PMR = 0.27, 95% CI 0.13–0.58), gastrointestinal tract (PMR = 0.49, 95% CI 0.32–0.76), respiratory system and intrathoracic organs (PMR = 0.20, 95% CI 0.07–0.57), soft tissues (PMR = 0.80, 95% CI 0.69–0.94) and urinary organs (PMR = 0.23, 95% CI 0.07–0.80) (Figure A5).

The comparison between North and the other Italian macro-regions (Centre, South and islands), reported in Figure A6, shows a certain degree of heterogeneity in the location of benign tumours.

Central Italian regions, when compared with Northern ones, showed significantly increased age-, breed- and neutering-status-adjusted risk of benign tumours of male sexual organs (PMR = 1.57, 95% CI 1.30–1.89), oral cavity and pharynx (PMR = 1.77, 95% CI 1.41–2.21) and soft tissues (PMR = 3.32, 95% CI 2.84–3.87), whereas there was a reduced risk of benign tumours of the mammary gland (PMR = 0.44, 95% CI 0.39–0.50) and of skin (PMR = 0.80, 95% CI 0.75–0.86).

South and Islands, when compared with Northern Italian regions, showed significantly increased age-, breed- and neutering-status-adjusted risks of benign tumours of bones, joints and cartilage (PMR = 2.50, 95% CI 1.02–6.12), eye, brain and meninges (PMR = 10.98, 95% CI 1.97–61.24), gastrointestinal tract (PMR = 3.04, 95% CI 1.95–4.74), male sexual organs (PMR = 1.39, 95% CI 1.07–1.79), oral cavity and pharynx (PMR = 1.70, 95% CI 1.26–2.31) and other female sexual organs (PMR = 1.73, 95% CI 1.20–2.51). South and Islands, when compared with Northern Italian regions, showed a reduced age-, breed- and neutering-status-adjusted risk of benign tumours of the mammary gland (PMR = 0.40, 95% CI 0.33–0.49).

The PM plotting of benign visceral tumours in different periods and macroregions showed a decrease in PMs in Northern Italy, a clear increase in PMs in Centre Italy and a smaller increase in south and islands (Figure A7). As the trends were not parallel, the effect of time was modified by the macroregion of residence.
